# 10-(1,2,2-Trichloro­vin­yl)-10*H*-pheno­thia­zine 5,5-dioxide

**DOI:** 10.1107/S160053681203245X

**Published:** 2012-07-21

**Authors:** Hideyuki Tabata, Tsunehisa Okuno

**Affiliations:** a Department of Material Science and Chemistry, Wakayama University, Sakaedani, Wakayama 640-8510, Japan

## Abstract

The title compound, C_14_H_8_Cl_3_NO_2_S, forms a dimeric structure by inter­molecular Cl⋯O=S inter­actions. The dimers make a two-dimensional array parallel to (101) by other Cl⋯O=S inter­actions. The two-dimensional network is found to be kept unchanged, although the trichloro­vinyl group is disordered (relative occupancies 0.65:0.35).

## Related literature

For related reviews of halogen bonding, see: Auffinger *et al.* (2004 ▶); Politzer *et al.* (2007 ▶). For related structures of phenothia­zine 5,5-dioxide compounds, see: Harrison *et al.* (2007 ▶); Kamtekar *et al.* (2011 ▶); Siddegowda *et al.* (2011**a* ▶,b* ▶); Zhu *et al.* (2007 ▶). For related structures with inter­molecular Cl⋯O=S contacts, see: Bandera *et al.* (2007 ▶); Choi *et al.* (2008 ▶); Douglas *et al.* (1993 ▶); Jovanovic *et al.* (1986 ▶). For the preparation of the title compound, see: Okuno *et al.* (2006 ▶).
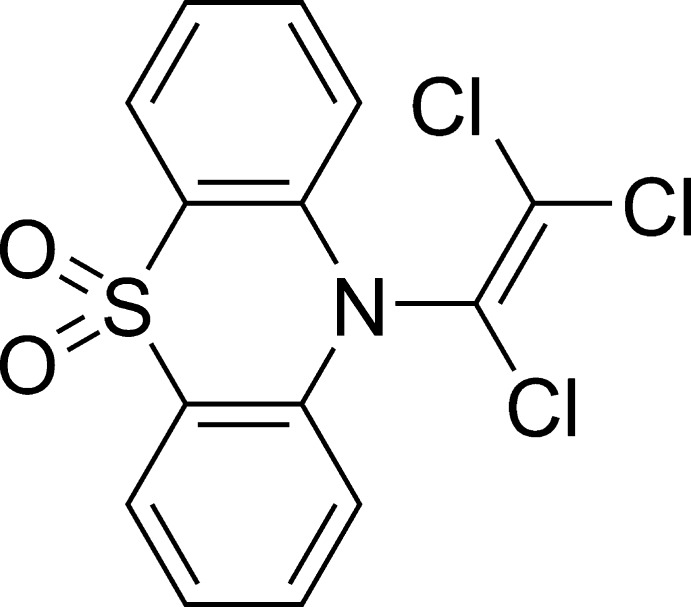


## Experimental

### 

#### Crystal data

C_14_H_8_Cl_3_NO_2_S
*M* *_r_* = 360.64
Monoclinic, 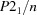

*a* = 7.703 (3) Å
*b* = 12.766 (5) Å
*c* = 14.884 (6) Å
β = 93.028 (6)°
*V* = 1461.7 (10) Å^3^
*Z* = 4
Mo *K*α radiation
μ = 0.77 mm^−1^
*T* = 93 K
0.10 × 0.10 × 0.10 mm

#### Data collection

Rigaku Saturn724+ diffractometer
Absorption correction: numerical (*NUMABS*; Rigaku, 1999 ▶) *T*_min_ = 0.964, *T*_max_ = 0.970
11979 measured reflections
3350 independent reflections
2660 reflections with *F* ^2^ > 2σ(*F* ^2^)
*R*_int_ = 0.040

#### Refinement

*R*[*F* ^2^ > 2σ(*F* ^2^)] = 0.040
*wR*(*F* ^2^) = 0.097
*S* = 1.07
3350 reflections
217 parameters
H-atom parameters constrained
Δρ_max_ = 0.38 e Å^−3^
Δρ_min_ = −0.34 e Å^−3^

### 

Data collection: *CrystalClear* (Rigaku, 2008 ▶); cell refinement: *CrystalClear*; data reduction: *CrystalClear*; program(s) used to solve structure: *SHELXS97* (Sheldrick, 2008 ▶); program(s) used to refine structure: *SHELXL97* (Sheldrick, 2008 ▶); molecular graphics: *ORTEP-3* (Farrugia, 1997 ▶); software used to prepare material for publication: *CrystalStructure* (Rigaku, 2010 ▶).

## Supplementary Material

Crystal structure: contains datablock(s) global, I. DOI: 10.1107/S160053681203245X/ff2076sup1.cif

Structure factors: contains datablock(s) I. DOI: 10.1107/S160053681203245X/ff2076Isup2.hkl

Supplementary material file. DOI: 10.1107/S160053681203245X/ff2076Isup3.cml

Additional supplementary materials:  crystallographic information; 3D view; checkCIF report

## Figures and Tables

**Table 1:** The geometry of inter­molecular Cl⋯O=S contacts (Å, °)

Atoms	Cl⋯O	C—Cl⋯O	Cl⋯O=S
C13*A*—Cl1⋯O1^i^=S1	3.1571 (19)	167.60 (14)	101.82 (9)
C14*B*—Cl1⋯O1^i^=S1	3.1571 (19)	157.5 (3)	101.82 (9)
C14*A*—Cl2⋯O1^ii^=S1	3.0521 (19)	175.00 (15)	166.90 (10)
C13*B*—Cl2⋯O1^ii^=S1	3.0521 (19)	144.7 (3)	166.90 (10)
C14*A*—Cl3*A*⋯O2^iii^=S1	3.174 (5)	157.7 (3)	100.61 (13)
C14*B*—Cl3*B*⋯O2^iii^=S1	3.175 (9)	160.8 (5)	104.74 (18)

Symmetry codes: (i) *x* + 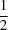, −*y* + 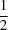, *z* − 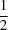; (ii) −*x*, −*y*, −*z* + 1; (iii) −*x* + 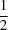, *y* − 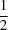, −*z* + 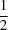.

**Fig. 1.**
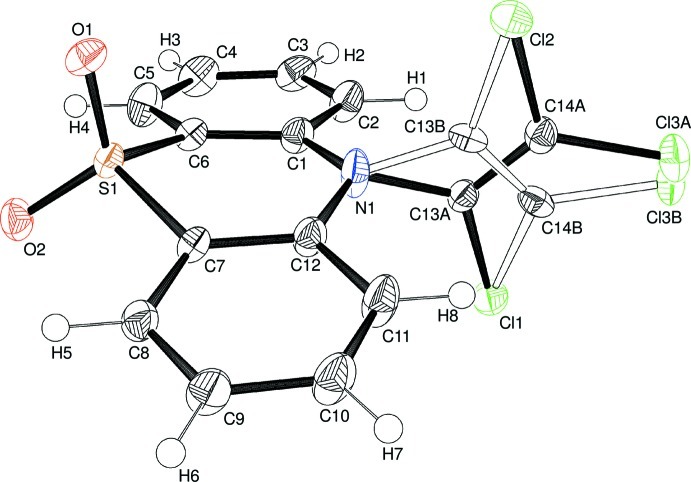
The asymmetric unit of the title compound with atom-numbering scheme. Displacement ellipsoids are drawn at the 50% probability level and H atoms are shown as small spheres. Disordered atoms are discriminated with A/B notation, and the minor disordered form is drawn as open bonds.

**Fig. 2.**
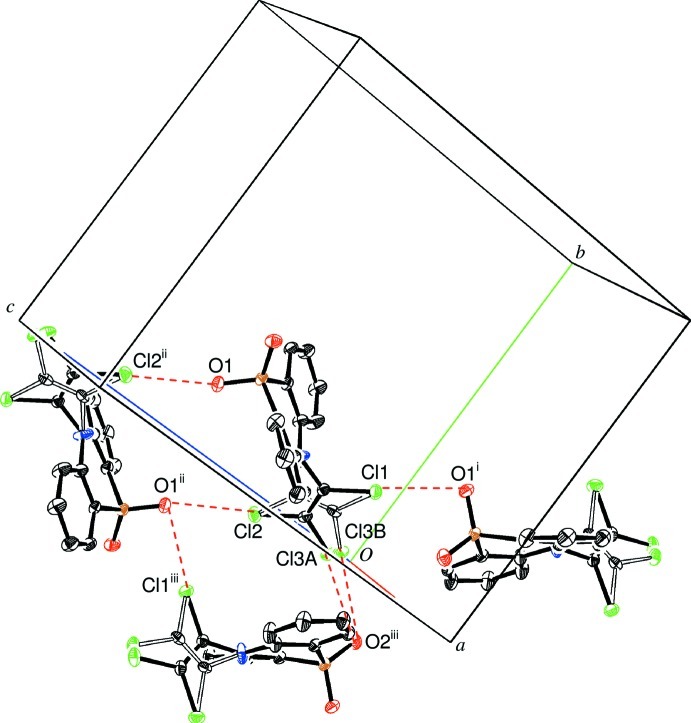
A view of the two-dimensional array on the (101) plane. Hydrogen atoms are omitted for clarity. The Cl···O=S interactions are shown as dashed lines. [Symmetry codes: (i) x + 1/2, -y + 1/2, z - 1/2; (ii) -x, -y, -z + 1; (iii) -x + 1/2, y - 1/2, -z + 1/2].

## supplementary crystallographic information

### Comment

Halogen bondings between halogen atoms and Lewis bases have been paid attention from the viewpoint of protein chemistry because of their use for the design of supramolecular assemblies (Auffinger *et al.* 2004; Politzer *et al.*, 2007). However, Cl···O=S interactions in heterocycles such as thiophenes and phenothiazines have been reported only in two cases (Douglas *et al.*, 1993; Jovanovic *et al.*, 1986). This is the first report of Cl···O=S interactions in phenothiazine 5,5-dioxide compounds.

The trichlorovinyl group is disordered to give two orientations, which are represented by the form A (C13A/C14A/Cl3A) and the form B (C13B/C14B/Cl3B) (Figure 1). The occupancies of A and B are determined to 0.65 for A and 0.35 for B as both disordered forms have similar thermal parameters. Both trichlorovinyl groups have a planar structure (the N1/C13A/C14A/Cl1/Cl2/Cl3A plane: r.m.s. deviation = 0.0465 Å and the N1/C13B/C14B/Cl1/Cl2/Cl3B plane: r.m.s. deviation = 0.0521 Å). The structures around N1 are pyramidal in A and planar in B, where the distances of N1 to the C1/C12/C13A plane and the C1/C12/C13B plane are 0.212 (2) Å and 0.071 (2) Å, respectively.

The phenothiazine moiety has a butterfly structure, where the dihedral angle between two benzene rings (the C1—C6 plane: r.m.s. deviation = 0.0103 Å and the C7—C12 plane: r.m.s. deviation = 0.0114 Å) is 162.51 (9)°. The central six-membered ring (the N1/C1/C6/S1/C7/C12 ring) has a boat form. The length of the equatorial S1—O2 bond is in the range of the reported values (1.4293 (14) Å - 1.4421 (11) Å) of phenothiazine 5,5-dioxide compounds (Harrison *et al.*, 2007; Kamtekar *et al.*, 2011; Siddegowda *et al.*, 2011**a*,b*; Zhu *et al.*, 2007), while the axial S1—O1 bond shows a longer bond length compared with that of the reported values (1.4294 (13) Å - 1.4495 (17) Å). This elongation may be caused by the intermolecular Cl···O=S interactions.

The molecules form a dimeric structure by the intermolecular Cl2^ii^···O1 interactions [Symmetry code: (ii) -*x*, -*y*, -*z* + 1]. The dimers make a two-dimensional array on the (101) plane by other Cl···O=S interactions (Figure 2). The intermolecular Cl···O=S distances are in the range of reported values (2.741 (3) Å - 3.267 (2) Å (Bandera *et al.*, 2007; Choi *et al.*, 2008)). Remarkable contacts cannot be observed between the arrays. The two-dimensional network is found to be kept unchanged, although the trichlorovinyl group is disordered.

### Experimental

The title compound was prepared according to a reported procedure (Okuno *et al.*, 2006). The single crystals with sufficient quality for X-ray analysis were obtained by concentration of a dichloromethane solution.

### Refinement

The occupancies of disordered trichlorovinyl groups were determined to 0.65 for A and 0.35 for B as both disordered forms have similar thermal parameters. All H atoms were placed at ideal positions and were treated as riding on their parent C atoms. *U*_iso_(H) values of the H atoms were set at 1.2*U*_eq_(parent atom).

### Crystal data

**Table:**

C_14_H_8_Cl_3_NO_2_S	*F*(000) = 728.00
*M**_r_* = 360.64	*D*_x_ = 1.639 Mg m^−^^3^
Monoclinic, *P*2_1_/*n*	Mo *K*α radiation, λ = 0.71075 Å
Hall symbol: -P 2yn	Cell parameters from 4473 reflections
*a* = 7.703 (3) Å	θ = 2.1–31.1°
*b* = 12.766 (5) Å	µ = 0.77 mm^−^^1^
*c* = 14.884 (6) Å	*T* = 93 K
β = 93.028 (6)°	Block, colourless
*V* = 1461.7 (10) Å^3^	0.10 × 0.10 × 0.10 mm
*Z* = 4	

### Data collection

**Table:**

Rigaku Saturn724+ diffractometer	2660 reflections with *F*^2^ > 2σ(*F*^2^)
Detector resolution: 7.111 pixels mm^-1^	*R*_int_ = 0.040
ω scans	θ_max_ = 27.5°
Absorption correction: numerical (*NUMABS*; Rigaku, 1999)	*h* = −9→9
*T*_min_ = 0.964, *T*_max_ = 0.970	*k* = −16→16
11979 measured reflections	*l* = −16→19
3350 independent reflections	

### Refinement

**Table:**

Refinement on *F*^2^	Secondary atom site location: difference Fourier map
*R*[*F*^2^ > 2σ(*F*^2^)] = 0.040	Hydrogen site location: inferred from neighbouring sites
*wR*(*F*^2^) = 0.097	H-atom parameters constrained
*S* = 1.07	*w* = 1/[σ^2^(*F*_o_^2^) + (0.0468*P*)^2^ + 0.463*P*] where *P* = (*F*_o_^2^ + 2*F*_c_^2^)/3
3350 reflections	(Δ/σ)_max_ < 0.001
217 parameters	Δρ_max_ = 0.38 e Å^−^^3^
0 restraints	Δρ_min_ = −0.34 e Å^−^^3^
Primary atom site location: structure-invariant direct methods	

### Special details

**Table:**

Refinement. Refinement was performed using all reflections. The weighted *R*-factor (*wR*) and goodness of fit (*S*) are based on *F*^2^. *R*-factor (gt) are based on *F*. The threshold expression of *F*^2^ > 2.0 σ(*F*^2^) is used only for calculating *R*-factor (gt).

### Fractional atomic coordinates and isotropic or equivalent isotropic displacement parameters (Å^2^)

**Table:**

	*x*	*y*	*z*	*U*_iso_*/*U*_eq_	Occ. (<1)
Cl1	0.35886 (7)	0.18970 (5)	0.16632 (4)	0.02359 (15)	
Cl2	0.04748 (7)	−0.04673 (5)	0.28388 (4)	0.02550 (15)	
Cl3A	0.1475 (6)	−0.0132 (4)	0.1010 (4)	0.0266 (6)	0.6500
Cl3B	0.1789 (11)	0.0075 (6)	0.0902 (6)	0.0245 (10)	0.3500
S1	0.22085 (7)	0.24593 (4)	0.51836 (3)	0.01767 (14)	
O1	0.1318 (2)	0.16939 (13)	0.57137 (11)	0.0228 (4)	
O2	0.2795 (2)	0.33968 (13)	0.56464 (11)	0.0249 (4)	
N1	0.2211 (3)	0.14499 (17)	0.33098 (13)	0.0280 (5)	
C1	0.3743 (3)	0.13499 (18)	0.38622 (15)	0.0215 (5)	
C2	0.5134 (3)	0.0742 (2)	0.35768 (16)	0.0280 (6)	
C3	0.6655 (3)	0.0672 (2)	0.40926 (17)	0.0284 (6)	
C4	0.6877 (3)	0.1205 (3)	0.49020 (17)	0.0292 (6)	
C5	0.5510 (3)	0.1783 (2)	0.52011 (17)	0.0278 (6)	
C6	0.3943 (3)	0.18481 (18)	0.46904 (14)	0.0188 (5)	
C7	0.0856 (3)	0.27768 (18)	0.42469 (14)	0.0177 (5)	
C8	−0.0368 (3)	0.35745 (19)	0.43520 (15)	0.0226 (5)	
C9	−0.1597 (4)	0.3786 (2)	0.36668 (16)	0.0277 (6)	
C10	−0.1595 (4)	0.3195 (3)	0.28853 (16)	0.0303 (6)	
C11	−0.0370 (3)	0.2424 (3)	0.27715 (16)	0.0296 (6)	
C12	0.0911 (3)	0.22094 (19)	0.34472 (15)	0.0220 (5)	
C13A	0.2397 (5)	0.1139 (3)	0.2389 (3)	0.0173 (7)	0.6500
C13B	0.1713 (9)	0.0641 (6)	0.2626 (5)	0.0179 (13)	0.3500
C14A	0.1559 (5)	0.0287 (3)	0.2101 (3)	0.0195 (7)	0.6500
C14B	0.2264 (9)	0.0852 (6)	0.1815 (6)	0.0189 (13)	0.3500
H1	0.5017	0.0377	0.3021	0.0336*	
H2	0.7574	0.0250	0.3892	0.0341*	
H3	0.7952	0.1173	0.5244	0.0351*	
H4	0.5636	0.2141	0.5760	0.0334*	
H5	−0.0352	0.3969	0.4894	0.0272*	
H6	−0.2428	0.4327	0.3730	0.0332*	
H7	−0.2457	0.3323	0.2418	0.0363*	
H8	−0.0397	0.2034	0.2227	0.0355*	

### Atomic displacement parameters (Å^2^)

**Table:**

	*U*^11^	*U*^22^	*U*^33^	*U*^12^	*U*^13^	*U*^23^
Cl1	0.0269 (3)	0.0239 (3)	0.0204 (3)	−0.0051 (3)	0.0049 (3)	0.0012 (3)
Cl2	0.0266 (3)	0.0235 (3)	0.0266 (3)	−0.0077 (3)	0.0025 (3)	0.0016 (3)
Cl3A	0.0265 (14)	0.0306 (15)	0.0225 (12)	0.0019 (9)	−0.0008 (9)	−0.0111 (10)
Cl3B	0.027 (3)	0.030 (3)	0.0172 (18)	−0.0011 (15)	0.0001 (15)	−0.0085 (16)
S1	0.0181 (3)	0.0219 (3)	0.0130 (3)	0.0017 (2)	−0.0001 (2)	−0.0001 (2)
O1	0.0223 (8)	0.0271 (9)	0.0195 (8)	0.0016 (7)	0.0046 (7)	0.0054 (7)
O2	0.0280 (9)	0.0260 (9)	0.0203 (9)	0.0004 (7)	−0.0038 (7)	−0.0062 (7)
N1	0.0294 (11)	0.0345 (12)	0.0189 (10)	0.0166 (10)	−0.0087 (9)	−0.0110 (9)
C1	0.0207 (12)	0.0249 (12)	0.0186 (11)	0.0061 (10)	−0.0016 (9)	0.0001 (10)
C2	0.0289 (13)	0.0355 (15)	0.0196 (12)	0.0152 (12)	0.0007 (10)	−0.0018 (11)
C3	0.0245 (13)	0.0330 (14)	0.0280 (13)	0.0108 (11)	0.0045 (11)	0.0066 (11)
C4	0.0171 (12)	0.0405 (16)	0.0297 (14)	0.0038 (11)	−0.0028 (10)	0.0041 (12)
C5	0.0207 (12)	0.0398 (16)	0.0226 (12)	−0.0012 (11)	−0.0019 (10)	−0.0028 (11)
C6	0.0177 (11)	0.0219 (12)	0.0166 (11)	0.0006 (9)	0.0009 (9)	0.0020 (9)
C7	0.0175 (11)	0.0223 (12)	0.0132 (10)	0.0006 (9)	0.0012 (9)	0.0014 (9)
C8	0.0257 (12)	0.0244 (12)	0.0181 (11)	0.0054 (10)	0.0043 (10)	−0.0009 (9)
C9	0.0273 (13)	0.0331 (14)	0.0227 (12)	0.0148 (11)	0.0006 (10)	0.0004 (11)
C10	0.0280 (13)	0.0413 (16)	0.0210 (12)	0.0129 (12)	−0.0047 (11)	−0.0012 (11)
C11	0.0278 (13)	0.0416 (16)	0.0187 (12)	0.0138 (12)	−0.0049 (10)	−0.0056 (11)
C12	0.0237 (12)	0.0251 (12)	0.0168 (11)	0.0077 (10)	−0.0020 (9)	−0.0016 (9)
C13A	0.0180 (18)	0.0191 (19)	0.0148 (19)	0.0027 (16)	0.0002 (15)	0.0002 (17)
C13B	0.016 (4)	0.012 (3)	0.026 (4)	0.000 (3)	0.002 (3)	0.002 (4)
C14A	0.0204 (19)	0.021 (2)	0.0177 (19)	0.0048 (16)	0.0016 (15)	−0.0027 (17)
C14B	0.016 (4)	0.013 (3)	0.027 (4)	0.001 (3)	−0.002 (3)	−0.001 (3)

### Geometric parameters (Å, º)

**Table:**

Cl1—C13A	1.747 (4)	C4—C5	1.379 (4)
Cl1—C14B	1.703 (7)	C5—C6	1.394 (3)
Cl2—C13B	1.745 (8)	C7—C8	1.402 (4)
Cl2—C14A	1.711 (4)	C7—C12	1.396 (4)
Cl3A—C14A	1.707 (7)	C8—C9	1.381 (4)
Cl3B—C14B	1.707 (11)	C9—C10	1.387 (4)
S1—O1	1.4509 (18)	C10—C11	1.380 (4)
S1—O2	1.4415 (18)	C11—C12	1.399 (4)
S1—C6	1.743 (3)	C13A—C14A	1.324 (6)
S1—C7	1.743 (3)	C13B—C14B	1.328 (11)
N1—C1	1.408 (3)	C2—H1	0.950
N1—C12	1.416 (4)	C3—H2	0.950
N1—C13A	1.441 (5)	C4—H3	0.950
N1—C13B	1.485 (8)	C5—H4	0.950
C1—C2	1.407 (4)	C8—H5	0.950
C1—C6	1.388 (4)	C9—H6	0.950
C2—C3	1.369 (4)	C10—H7	0.950
C3—C4	1.387 (4)	C11—H8	0.950
			
O1—S1—O2	116.39 (10)	C7—C12—C11	117.3 (3)
O1—S1—C6	108.83 (11)	Cl1—C13A—N1	121.1 (3)
O1—S1—C7	108.16 (11)	Cl1—C13A—C14A	121.2 (4)
O2—S1—C6	110.19 (11)	N1—C13A—C14A	117.6 (4)
O2—S1—C7	110.36 (11)	Cl2—C13B—N1	124.3 (5)
C6—S1—C7	101.91 (11)	Cl2—C13B—C14B	122.2 (6)
C1—N1—C12	123.7 (2)	N1—C13B—C14B	113.5 (6)
C1—N1—C13A	114.1 (3)	Cl2—C14A—Cl3A	116.0 (3)
C1—N1—C13B	121.0 (4)	Cl2—C14A—C13A	120.1 (4)
C12—N1—C13A	115.6 (3)	Cl3A—C14A—C13A	123.9 (4)
C12—N1—C13B	114.6 (3)	Cl1—C14B—Cl3B	117.0 (6)
N1—C1—C2	120.1 (2)	Cl1—C14B—C13B	120.1 (6)
N1—C1—C6	121.8 (2)	Cl3B—C14B—C13B	122.8 (7)
C2—C1—C6	118.0 (2)	C1—C2—H1	119.668
C1—C2—C3	120.7 (3)	C3—C2—H1	119.665
C2—C3—C4	121.1 (3)	C2—C3—H2	119.434
C3—C4—C5	118.8 (3)	C4—C3—H2	119.419
C4—C5—C6	120.6 (3)	C3—C4—H3	120.585
S1—C6—C1	121.81 (17)	C5—C4—H3	120.590
S1—C6—C5	117.24 (18)	C4—C5—H4	119.689
C1—C6—C5	120.6 (2)	C6—C5—H4	119.683
S1—C7—C8	117.16 (17)	C7—C8—H5	120.053
S1—C7—C12	121.16 (18)	C9—C8—H5	120.047
C8—C7—C12	121.5 (2)	C8—C9—H6	120.592
C7—C8—C9	119.9 (3)	C10—C9—H6	120.585
C8—C9—C10	118.8 (3)	C9—C10—H7	119.271
C9—C10—C11	121.5 (3)	C11—C10—H7	119.270
C10—C11—C12	120.8 (3)	C10—C11—H8	119.575
N1—C12—C7	122.1 (2)	C12—C11—H8	119.581
N1—C12—C11	120.5 (2)		
			
O1—S1—C6—C1	87.90 (18)	C13B—N1—C12—C11	−24.3 (5)
O1—S1—C6—C5	−85.66 (17)	N1—C1—C2—C3	177.5 (2)
O1—S1—C7—C8	86.33 (17)	N1—C1—C6—S1	10.3 (4)
O1—S1—C7—C12	−89.26 (17)	N1—C1—C6—C5	−176.39 (19)
O2—S1—C6—C1	−143.36 (16)	C2—C1—C6—S1	−170.48 (19)
O2—S1—C6—C5	43.07 (18)	C2—C1—C6—C5	2.9 (4)
O2—S1—C7—C8	−42.04 (18)	C6—C1—C2—C3	−1.8 (4)
O2—S1—C7—C12	142.37 (15)	C1—C2—C3—C4	−0.9 (4)
C6—S1—C7—C8	−159.07 (15)	C2—C3—C4—C5	2.4 (4)
C6—S1—C7—C12	25.34 (19)	C3—C4—C5—C6	−1.3 (4)
C7—S1—C6—C1	−26.21 (19)	C4—C5—C6—S1	172.3 (2)
C7—S1—C6—C5	160.23 (15)	C4—C5—C6—C1	−1.4 (4)
C1—N1—C12—C7	−14.0 (4)	S1—C7—C8—C9	−173.12 (15)
C1—N1—C12—C11	165.5 (2)	S1—C7—C12—N1	−8.8 (3)
C12—N1—C1—C2	−166.01 (19)	S1—C7—C12—C11	171.66 (14)
C12—N1—C1—C6	13.2 (4)	C8—C7—C12—N1	175.7 (2)
C1—N1—C13A—Cl1	−71.3 (4)	C8—C7—C12—C11	−3.7 (4)
C1—N1—C13A—C14A	112.1 (3)	C12—C7—C8—C9	2.5 (4)
C13A—N1—C1—C2	−15.9 (4)	C7—C8—C9—C10	0.3 (4)
C13A—N1—C1—C6	163.4 (3)	C8—C9—C10—C11	−1.7 (4)
C1—N1—C13B—Cl2	88.4 (6)	C9—C10—C11—C12	0.4 (4)
C1—N1—C13B—C14B	−93.3 (5)	C10—C11—C12—N1	−177.2 (3)
C13B—N1—C1—C2	24.4 (5)	C10—C11—C12—C7	2.3 (4)
C13B—N1—C1—C6	−156.4 (4)	Cl1—C13A—C14A—Cl2	177.4 (2)
C12—N1—C13A—Cl1	81.4 (3)	Cl1—C13A—C14A—Cl3A	−2.6 (5)
C12—N1—C13A—C14A	−95.2 (4)	N1—C13A—C14A—Cl2	−6.0 (5)
C13A—N1—C12—C7	−163.7 (3)	N1—C13A—C14A—Cl3A	174.0 (3)
C13A—N1—C12—C11	15.8 (4)	Cl2—C13B—C14B—Cl1	−175.6 (4)
C12—N1—C13B—Cl2	−82.2 (6)	Cl2—C13B—C14B—Cl3B	1.1 (9)
C12—N1—C13B—C14B	96.1 (5)	N1—C13B—C14B—Cl1	6.0 (8)
C13B—N1—C12—C7	156.3 (4)	N1—C13B—C14B—Cl3B	−177.2 (4)

### The geometry of intermolecular Cl···O=S contacts (Å, °).

**Table:**

Atoms	Cl···O	C-Cl···O	Cl···O=S
C13A-Cl1···O1^i^=S1	3.1571 (19)	167.60 (14)	101.82 (9)
C14B-Cl1···O1^i^=S1	3.1571 (19)	157.5 (3)	101.82 (9)
C14A-Cl2···O1^ii^=S1	3.0521 (19)	175.00 (15)	166.90 (10)
C13B-Cl2···O1^ii^=S1	3.0521 (19)	144.7 (3)	166.90 (10)
C14A-Cl3A···O2^iii^=S1	3.174 (5)	157.7 (3)	100.61 (13)
C14B-Cl3B···O2^iii^=S1	3.175 (9)	160.8 (5)	104.74 (18)

Symmetry codes: (i) *x* + 1/2, -*y* + 1/2, *z* - 1/2; (ii) -*x*, -*y*, -*z* + 1; (iii) -*x* + 1/2, *y* - 1/2, -*z* + 1/2.
